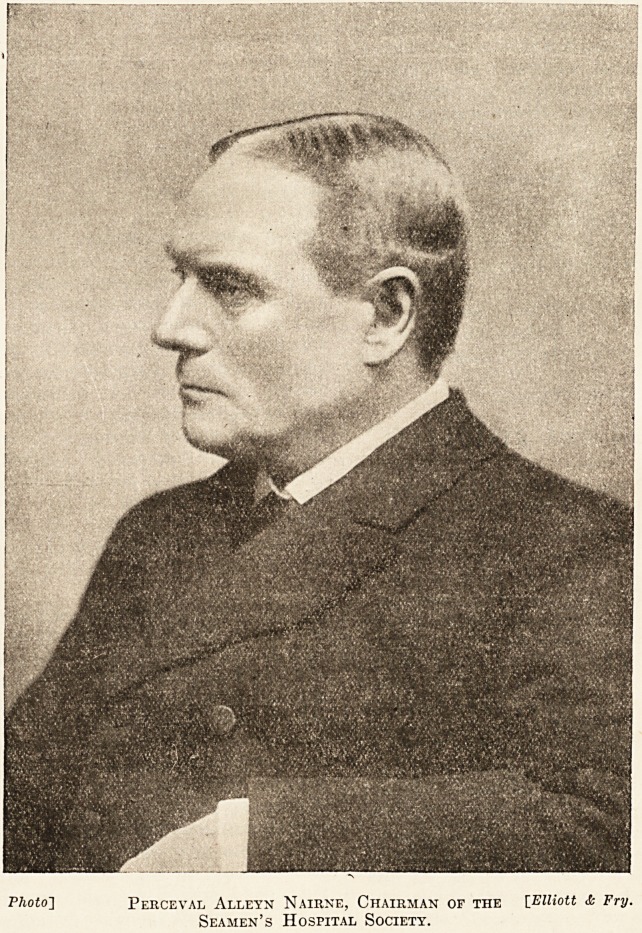# Eminent Chairmen Series
*The previous articles in this series appeared in The Hospital of Oct. 1, Nov. 1, Dec. 10, Jan. 14, Feb. 11, and March 11.


**Published:** 1911-04-22

**Authors:** 


### SPECIAL INSTITUTIONAL ARTICLE.

### EMINENT CHAIRMEN SERIES.*

### VII.—PERCEVAL ALLEYNE NAIRNE, Chairman of the Seamen's Hospital Society.

Mr. Perceval Alleyne Nairne is probably the doyen of Hospital Committee-men, for he has served on the Committee of the Corporation of the Seamen's Hospital Society since the year 1869, when he succeeded his father, the late Captain H. C. Nairne.

Captain Nairne (who fought at Copenhagen in 1801, on which occasion he served with Franklin), was a member of the Committee of Management of the Seamen's Hospital Society from 1836 to 1867. Captain Nairne was not the only relation of the present Chairman who sat upon the Committee of the "Dreadnought," for among those who have guided the destiny of the Charity through the nineteenth century are to be found many members of his family. Mr. Nairne's grandfather, Nathaniel Domett, served from 1835 to 1848, and his uncle, John Domett, from 1870 to 1883. Another of his relations is Sir Frederick Young, K.C.M.G., a member of the Committee from 1892 to 1909, and now a vice-president. Sir Frederick Young is ninety-four years of age, and the pioneer of Colonial Federation. Admiral William Young, grandfather of Sir Frederick Young, and great-uncle of Mr. P. A. Nairne, served from 1821 to 1844, when he was elected a vice-president. His name is mentioned in the Act of Incorporation passed in 1833. Mr. George Frederick Young, M.P., Sir Frederick's father and Mr. Nairne's uncle, also served on the Board. Mr. Nairne has had many other distinguished colleagues during the last forty-two years—a list too long to mention here.

Photo] Perceval Alleyn Nairne, Chairman of the Seamen's Hospital Society. [*Elliott & Fry.*

#### Deputy-Chairman of the Dreadnought.

On the appointment of Sir Frederick Cleeve, K.C.B., R.N., as Chairman in 1886, Mr. Nairne was promoted to be deputy-chairman, and held this office until the year 1895. In this year Admiral the Hon. Francis Egerton, who had been elected Chairman in the interim, died, and the committee unanimously resolved to invite Mr. Nairne to take the chair. Although he and his family had been connected with the Society since its foundation, he declined to occupy the position, on the ground that he was not a sailor or a shipowner.

The Board abstained for two years from electing anyone chairman, the duties being carried on by Mr. Nairne, the deputy-chairman.

However, in 1898 Mr. Nairne acceded to the wish of his colleagues, and assumed the chair, which he holds to this day.

#### The Evolution of the Dreadnought Hospital.

During Mr. Nairne's services the Seamen's Hospital Society has seen greater changes and expansions than in any previous period of the history of the Charity, and probably greater advancement than any other institution in a similar time. When

* The previous articles in this series appeared in The Hospital of Oct. 1, Nov. 1, Dec. 10, Jan. 14, Feb. 11, and March 11.

Mr. Nairne joined the Board the "Dreadnought" lay in the River Thames. She was an old three-decker called the "Caledonia," which had been renamed the "Dreadnought" after her predecessor. There was accommodation for about 200 sailors, and there was no out-patient department. In 1870, on Greenwich Hospital being closed as a Home for Pensioners, the Admiralty offered to the Society the Infirmary of Greenwich Hospital, in which building the main work of the Society is now carried on.

In the year 1875 the committee secured the services of Mr. H. C. Burdett, whose untiring energy helped to give that impulse to the Charity which it has maintained ever since.

In 1881 the first branch of the Society was opened by the establishment of the Dispensary in the East End of London.

In 1887 the Dispensary at Gravesend was opened.

In 1890 the branch hospital was opened in the dock district, with twelve beds and an out-patient department.

The School of Tropical Medicine.

In 1899 came the most successful School of Medicine probably ever inaugurated—the London School of Tropical Medicine, while at the same time the Albert Dock Hospital was enlarged to fifty-two beds.

In 1905 was established the London School of Clinical Medicine.

This but roughly gives an idea of what has been accomplished since Mr. Nairne joined the Committee. The income of the Charity has doubled, while the number of patients treated has increased from 2,000 to 34,000.

These are not, however, the only activities of Mr. Nairne. He is a solicitor with a large practice, but he yet finds time not only to control the work of the Seamen's Hospital Society but to occupy the position of lay secretary to the Rochester Diocesan Conference, which, on the establishment of the See of Southwark became the Southwark Diocesan Conference. He is still the moving spirit in that organisation.

The work that is carried on by the Dreadnought Hospital at Greenwich is of a specialised kind. In the first place, it is a hospital for men; secondly, it is, so far as in-patients are concerned, exclusively a hospital for seamen.

On more than one occasion we have pointed out the special claims which such an institution, carrying out such special work, has on the generosity of the general public. We owe much to our seamen, and we do comparatively little to show our fellow-feeling with them, particularly in this large metropolis of ours, where the average landsman, notwithstanding the fact that London is one of the largest seaports in the world, so rarely comes into contact with the distressed sailor. That is one reason why the Dreadnought should appeal to every section of the public. It is the most cosmopolitan hospital in England; it is one of the best-managed; it is one that enjoys the almost unique distinction, so far as London is concerned, of having secured the hearty co-operation between the hospital authorities and staff and the general practitioners in its district. In addition, it is the home of one of the largest post-graduate medical schools in the world, and has associated with it a school of Tropical Medicine that has gained a world-wide reputation through the excellence of the work which it has carried out.

The Chairman has taken a great personal interest in the hospital and the school. The latter, it is safe to say, is one of the finest post-graduate institutions in the world: it already possesses the deserved reputation of being the best school for operative surgery in London; students from all the other medical schools go there for their special work in this subject preliminary to the final F.R.C.S. or M.S. examinations. The School of Tropical Medicine has only one rival to it in this country, the Liverpool School, which, through the generosity and munificence of the late Sir Alfred Jones, has been enabled to outstrip the Greenwich institution with regard to expeditionary work. But as a school, and apart from a research institution, the latter has, perhaps, greater claims for public support than the Liverpool school. It has instructed many hundreds of medical men and officials from all parts of the world, and is doing what may really be called an Imperial work in the best sense of the term. What it wants is the active interest and staunch support of an Imperialist of the stamp of the late Sir Alfred Jones. It is desirable that London should have a great school for the study of Tropical Medicine, and the claims of the Dreadnought London School to be the nucleus of such an institution are too great to be disregarded.

We have recently (The Hospital, April 1, p. 15) referred to the action which the Greenwich Hospital has taken in the matter of limiting out-patient attendances and thereby combating hospital abuse. The method then described has been found to work well, and the out-patient department at the Dreadnought, although it is not yet ideal, is much more a consultative and casualty department than is the case in the majority of London hospitals. No doubt the method of ticket admittances is as yet too novel, perhaps too revolutionary, for the general practitioner to understand, and it must be remembered that the district in which the hospital stands is drained, among other institutions, by hospitals like Shadwell, Guy's, St. Thomas', Evelina, and the Belgrave, which have as yet made no attempt to imitate the Dreadnought's example, though most of them possess almoners and have endeavoured to cope with the problem of hospital abuse in some way or other.

The Dreadnought has always been eager to profit by the latest developments in the field of medical treatment and diagnosis. It was one of the first London hospitals to establish a thorough Roentgen-ray cabinet; it has been one of the first in the field with a proper serological department, which is excellently equipped and does excellent work in a quiet, unostentatious way. Vaccine treatment, blood examinations, and the mass of work now included in the syllabus of such a department are attended to not only for the in and out patient departments, but for private practitioners as well, who are loud in their praises of the reliability and promptness with which the work is executed. At a time when other hospitals made capital by judicious advertising out of the fact that they were experimenting on a minute scale with novelties such as spinal anæsthesia and "606," the staff at the Dreadnought was testing these new aids as a matter of routine, and carefully collating its evidence, with the result that the work done at this institution has come to be accepted as authoritative.

In Private Life.

It was hardly necessary for Mr. Nairne to decline the office of Chairman of the Seamen's Hospital Society on the ground that he was not a sailor, for he has been a yachtsman throughout his life and is now, and has been for many years, the owner of the schooner "Feronia." On that vessel he extends throughout the summer months hospitality to his many friends, among whom he is courteous enough to include some of those in his own employ, and at the hospital.

Mr. Nairne is a bachelor, and still lives amid the surroundings of his parents in the old-world suburb of Denmark Hill. There he holds an almost open house to young men, many of them kinsmen of his own. His quiet and retiring demeanour would at first sight hardly lead a casual observer to realise his controlling power. Chairman of a committee which is composed of able and assertive spirits (for a large portion of the committee consists of members of the Senior Service), he exercises a remarkable influence. His judgment seems unerring, his arguments, put quietly and considerately, are convincing in a very exceptional degree. His personality has an effect upon his colleagues and upon the Society which has made the "Dreadnought" what it is, while he is looked up to by the staff and by all the employees of the Charity with a feeling of confidence that enables everyone concerned to realise the justice of the ruling of the Board.

It is to such a man as this that the success and unprecedented expansion of the work of the Seamen's Hospital Society is due.

## Figures and Tables

**Figure f1:**